# Observations on Fungus Hæmatodes

**Published:** 1808-05

**Authors:** 


### *To the Editors of the Medical and Phyſical Journal.*

Gentlemen,

As in the momentary quarrels of friends, the person who interferes, either impartially to conciliate, or particularly to assist, is seldom looked upon as a friend; or at least, is seldom thanked by either party: so, perhaps, in the disputes of your correspondents, it would be proper to leave the parties to decide them as they please. But as there

---

there is among mankind, a general inclination or desire to attack presumptuous ignorance, or arrogant vanity, wherever they appear, even although these have in their effects no relation to the individual who makes the attack; and as in the present case the subject of dispute is one that concerns our whole profession, and consequently me as an individual member of it, I may be forgiven for intruding upon the present discussion. In your last number is a paper by Mr. Weaver, in which he attempts to delineate the true nature of Fungus Hæmatodes. He commences by stating some vague arguments, in order to confute the opinion of Mr. Shearly of the analogy between Fungus Hæmatodes and Cancer. As I have not the paper of Mr. Shearly by me, and, indeed, as by some accident I neglected to peruse his cases, I can say nothing on the merits of the question on either side: although I think Mr. Weaver has clearly proven, that there is not the smallest affinity between Cancer and *his* Fungus Hæmatodes.

Though, perhaps, it is not so "well known" as Mr. W. imagines, that small-pox produces cancer, although we could, perhaps, find arguments strong as Mr. W's to advance against that opinion; and although we could produce many cases of cancer, in persons whose families were never suspected to be scrophulous; yet we shall say nothing on the subject, but proceed to consider the remainder of the paper.

When Mr. W. says, "I do not recollect that modern surgery affords us this disease under its present Grecian dress, "fungus αἱματώδης;" does he mean that he never saw the name of the disease written, till he wrote it forsooth, in Greek characters? and does he mean to insinuate that because surgeons have forborne to make use of a *nosology* partly Greek and partly Latin, that they are unacquainted with the signification of *hæmatodes*, and *consequently*, that (according to the decision of his *soi-disant* tribunal) they are ignorant of the disease? If such is his meaning, and if upon the strength of this brilliant discovery of his, he has the temerity to hold up to us a well-known and simple affection, for one of the most terrible diseases 'that flesh is heir to:' instead of complimenting him on his acumen and classic abilities, we would recommend to him "a perusal of some late nosological authors," which "might tend to illuminate his mind."

Mr. Hey and Mr. Burns have given us correct and melancholy descriptions of this dreadful disease: such descriptions, indeed, as must lead every one who has seen them to to conclude, either that Mr. Weaver never has perused them, or that it is his intention to give an account of a disease totally different from that affection named by them Fungus Hæmatodes, or Spongoid Inflammation. The disease which medical men have been accustomed to treat under this appellation, is one of a specific nature, having actions peculiar to itself; commencing in a small tumour somewhat of the appearance of a varix, gradually increasing in size and inveteracy, involving in its progress, and *as it were*, destroying the neighbouring parts. This tumour, if left to itself, bursts, and a fungus protrudes, supposed to be coagulable lymph organized: and, indeed, its appearance, and its astonishing tendency to hæmorrhagy, prove this. The tumour in its earliest stages can sometimes be extirpated with success; but in its more advanced progress, amputation alone, (and too frequently not even that,) can be of any avail. Now, does the disease described by Mr. W. bear the least resemblance to this? In fact, he is so sparing in his information, in the case of *Lees*, that we are, for the most part, left to conjecture. But from the manner he received it, from the extent and suddenness of appearance of the tumour, it is evident that it must have been an effusion into the cellular substance; and from the event of the case, we are led to know that the extravasation was sanguineous. A disease neither new nor singular: for who, in every week of his practice, does not meet with extravasation of blood? an affection confessedly as "*diametrically opposite to cancer*," as ague and hooping-cough! But, what? aye, but if from some cause the blood is not absorbed; if it coagulates, and remains for some time a lifeless mass; and if from the exertions of the system it becomes at last about to be evacuated; why then it must assume the sounding and formidable title of *Fungus Hæmatodes!* and verily Mr. Weaver has given a most characteristic epithet to *his fungus*; for 'tis not only αἱματώδης, but good pure αἷμα itself.

We have all found cases like that described by Mr. Hey, and also like that described by Mr. Weaver; but did we ever think of classing them under the same head, or consider them of equal danger, or requiring the same treatment? Should we ever have thought of amputating a limb for a simple effusion? or stuffing arsenic into a lifeless mass of coagulated blood? Assuredly not. Mr. W. does not specify the time between the accident and the protrusion of the fungus: but he certainly acted very properly in enlarging the opening, though in so doing he only did what what *every medical Tyro* would have done, even although he had never heard of Hippocrates or his "Nϰσων φυσις ιαlgος." We are not informed by Mr. W. whether there was any hemorrhagy from the fungus: he *waves* this information: by so doing, I think he pays a greater compliment to the understanding of his readers, than he does in many other particulars of intelligence in his paper. For upon hearing a description of a coagulum *of blood*, we will not be very apt to ask if there was any hæmorrhage from it.

But, perhaps, I am attacking Mr. W. on a point which he does not want to enter upon. Perhaps he is not serious in his opinions, and merely wrote the paper in question, to shew his acquaintance with *belles lettres;* to let the world know that he has not yet altogether forgot his Latin; and that he is still in possession of that antient lexicon that used to befriend his Greek-puzzled noddle in the days of his youth. In some corner of the said lexicon, he perhaps may find the old saw of Solon, "Γνῶθι σεαυτον," the which I would advise him to keep within range of his optics, the next time he addresses the public. For let him be assured, that though shreds of Greek and Latin may look very fine in the eye of ignorance, they will never uphold a "baseless fabric," or turn the tide of opinion in his favour, if common sense, in common or in homely language, declare against him.

*His Majesty's Ship ———, at Sea,* F. G.

*December* 19, 1807.

